# Supplementary material for: Venomix: a simple bioinformatic pipeline for identifying and characterizing toxin gene candidates from transcriptomic data
Source: PeerJ. 2018 Jul 31;6:e5361. doi: 10.7717/peerj.5361 (PMC6074769; doi:10.7717/peerj.5361)
Supplement: Supplemental Information 3 [file peerj-06-5361-s003.gz › FinalOutput_E-20/Thrombin-like_enzyme_TLBan_1/finaltree.pdf]

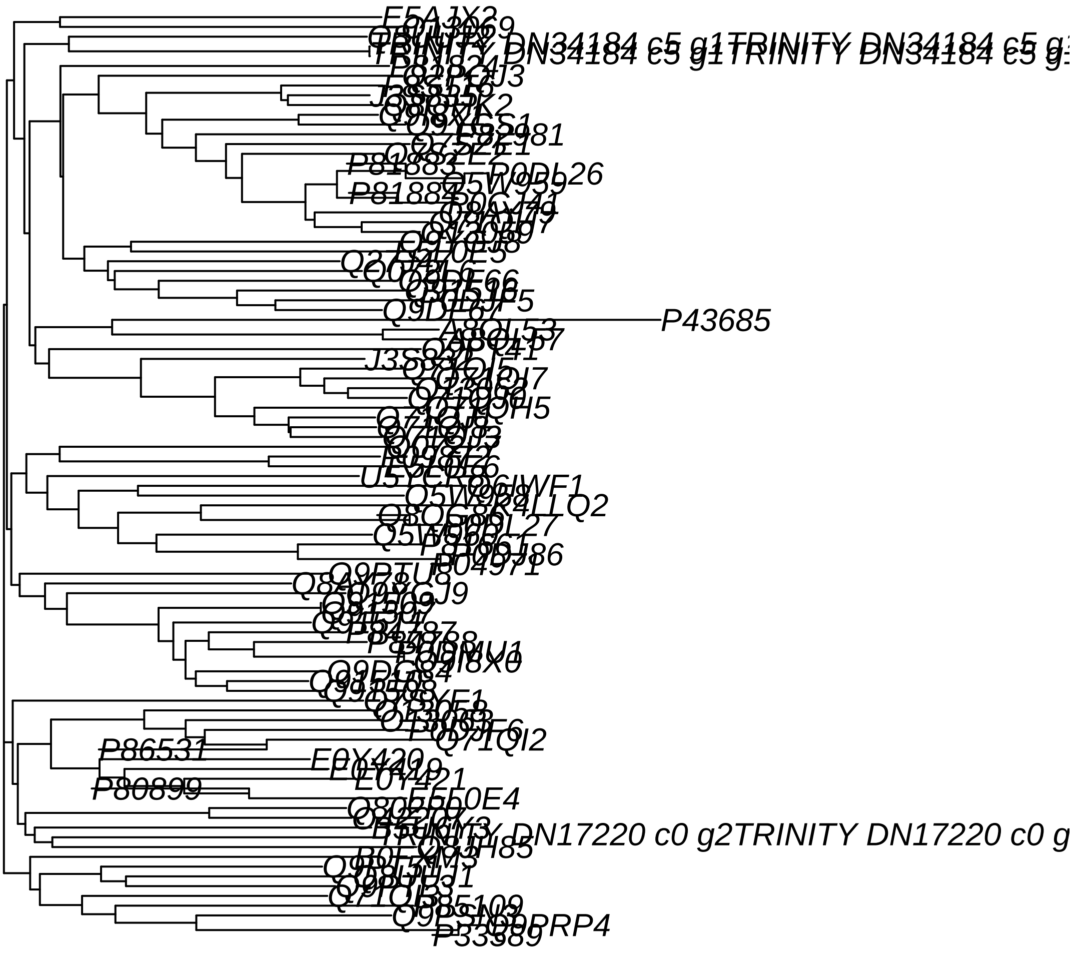

E5A1X2
P43685
P86531
P80899
E0Y420
L0Y421
E5S0E4
Q7TQI2
P33389
